# Supplementary material for: Resolving the full spectrum of human genome variation using Linked-Reads
Source: Genome Res. 2019 Apr;29(4):635–45. doi: 10.1101/gr.234443.118 (PMC6442396; doi:10.1101/gr.234443.118)
Supplement: Supplemental Material [file supp_gr.234443.118_Supplemental_longranger-2.2.zip › longranger-2.2.tar.bz2!/lr2.2-public/lib/stan/cmdstan/stan/doc/api/html/classstan_1_1mcmc_1_1adapt__dense__e__static__hmc-members.html]

Stan: Member List


|  |  |
| --- | --- |
|  | Stan  2.5.0  probability, sampling & optimization |


- Main Page
- Related Pages
- Namespaces
- Classes
- Files

- Class List
- Class Index
- Class Hierarchy
- Class Members

All Classes Namespaces Files Functions Variables Typedefs Enumerations Enumerator Friends Macros Pages

- stan
- mcmc
- adapt\_dense\_e\_static\_hmc

stan::mcmc::adapt\_dense\_e\_static\_hmc< M, BaseRNG > Member List

This is the complete list of members for stan::mcmc::adapt\_dense\_e\_static\_hmc< M, BaseRNG >, including all inherited members.

|  |  |  |
| --- | --- | --- |
| adapt\_dense\_e\_static\_hmc(M &m, BaseRNG &rng, std::ostream \*o=&std::cout, std::ostream \*e=0) | stan::mcmc::adapt\_dense\_e\_static\_hmc< M, BaseRNG > | inline |
| adapt\_flag\_ | stan::mcmc::base\_adapter | protected |
| adapting() | stan::mcmc::base\_adapter | inline |
| base\_adapter() | stan::mcmc::base\_adapter | inline |
| base\_hmc(M &m, BaseRNG &rng, std::ostream \*o, std::ostream \*e) | stan::mcmc::base\_hmc< M, dense\_e\_point, dense\_e\_metric, expl\_leapfrog, BaseRNG > | inline |
| base\_mcmc(std::ostream \*o, std::ostream \*e) | stan::mcmc::base\_mcmc | inline |
| base\_static\_hmc(M &m, BaseRNG &rng, std::ostream \*o, std::ostream \*e) | stan::mcmc::base\_static\_hmc< M, dense\_e\_point, dense\_e\_metric, expl\_leapfrog, BaseRNG > | inline |
| covar\_adaptation\_ | stan::mcmc::stepsize\_covar\_adapter | protected |
| dense\_e\_static\_hmc(M &m, BaseRNG &rng, std::ostream \*o=&std::cout, std::ostream \*e=0) | stan::mcmc::dense\_e\_static\_hmc< M, BaseRNG > | inline |
| disengage\_adaptation() | stan::mcmc::adapt\_dense\_e\_static\_hmc< M, BaseRNG > | inlinevirtual |
| engage\_adaptation() | stan::mcmc::base\_adapter | inlinevirtual |
| epsilon\_ | stan::mcmc::base\_hmc< M, dense\_e\_point, dense\_e\_metric, expl\_leapfrog, BaseRNG > | protected |
| epsilon\_jitter\_ | stan::mcmc::base\_hmc< M, dense\_e\_point, dense\_e\_metric, expl\_leapfrog, BaseRNG > | protected |
| err\_stream\_ | stan::mcmc::base\_mcmc | protected |
| get\_covar\_adaptation() | stan::mcmc::stepsize\_covar\_adapter | inline |
| get\_current\_stepsize() | stan::mcmc::base\_hmc< M, dense\_e\_point, dense\_e\_metric, expl\_leapfrog, BaseRNG > | inline |
| get\_L() | stan::mcmc::base\_static\_hmc< M, dense\_e\_point, dense\_e\_metric, expl\_leapfrog, BaseRNG > | inline |
| get\_nominal\_stepsize() | stan::mcmc::base\_hmc< M, dense\_e\_point, dense\_e\_metric, expl\_leapfrog, BaseRNG > | inline |
| get\_sampler\_diagnostic\_names(std::vector< std::string > &model\_names, std::vector< std::string > &names) | stan::mcmc::base\_hmc< M, dense\_e\_point, dense\_e\_metric, expl\_leapfrog, BaseRNG > | inlinevirtual |
| get\_sampler\_diagnostics(std::vector< double > &values) | stan::mcmc::base\_hmc< M, dense\_e\_point, dense\_e\_metric, expl\_leapfrog, BaseRNG > | inlinevirtual |
| get\_sampler\_param\_names(std::vector< std::string > &names) | stan::mcmc::base\_static\_hmc< M, dense\_e\_point, dense\_e\_metric, expl\_leapfrog, BaseRNG > | inlinevirtual |
| get\_sampler\_params(std::vector< double > &values) | stan::mcmc::base\_static\_hmc< M, dense\_e\_point, dense\_e\_metric, expl\_leapfrog, BaseRNG > | inlinevirtual |
| get\_stepsize\_adaptation() | stan::mcmc::stepsize\_covar\_adapter | inline |
| get\_stepsize\_jitter() | stan::mcmc::base\_hmc< M, dense\_e\_point, dense\_e\_metric, expl\_leapfrog, BaseRNG > | inline |
| get\_T() | stan::mcmc::base\_static\_hmc< M, dense\_e\_point, dense\_e\_metric, expl\_leapfrog, BaseRNG > | inline |
| hamiltonian\_ | stan::mcmc::base\_hmc< M, dense\_e\_point, dense\_e\_metric, expl\_leapfrog, BaseRNG > | protected |
| init\_stepsize() | stan::mcmc::base\_hmc< M, dense\_e\_point, dense\_e\_metric, expl\_leapfrog, BaseRNG > | inline |
| integrator\_ | stan::mcmc::base\_hmc< M, dense\_e\_point, dense\_e\_metric, expl\_leapfrog, BaseRNG > | protected |
| L\_ | stan::mcmc::base\_static\_hmc< M, dense\_e\_point, dense\_e\_metric, expl\_leapfrog, BaseRNG > | protected |
| name() | stan::mcmc::base\_mcmc | inline |
| name\_ | stan::mcmc::base\_mcmc | protected |
| nom\_epsilon\_ | stan::mcmc::base\_hmc< M, dense\_e\_point, dense\_e\_metric, expl\_leapfrog, BaseRNG > | protected |
| out\_stream\_ | stan::mcmc::base\_mcmc | protected |
| rand\_int\_ | stan::mcmc::base\_hmc< M, dense\_e\_point, dense\_e\_metric, expl\_leapfrog, BaseRNG > | protected |
| rand\_uniform\_ | stan::mcmc::base\_hmc< M, dense\_e\_point, dense\_e\_metric, expl\_leapfrog, BaseRNG > | protected |
| sample\_stepsize() | stan::mcmc::base\_hmc< M, dense\_e\_point, dense\_e\_metric, expl\_leapfrog, BaseRNG > | inline |
| seed(const Eigen::VectorXd &q) | stan::mcmc::base\_hmc< M, dense\_e\_point, dense\_e\_metric, expl\_leapfrog, BaseRNG > | inline |
| set\_nominal\_stepsize(const double e) | stan::mcmc::base\_static\_hmc< M, dense\_e\_point, dense\_e\_metric, expl\_leapfrog, BaseRNG > | inlinevirtual |
| set\_nominal\_stepsize\_and\_L(const double e, const int l) | stan::mcmc::base\_static\_hmc< M, dense\_e\_point, dense\_e\_metric, expl\_leapfrog, BaseRNG > | inline |
| set\_nominal\_stepsize\_and\_T(const double e, const double t) | stan::mcmc::base\_static\_hmc< M, dense\_e\_point, dense\_e\_metric, expl\_leapfrog, BaseRNG > | inline |
| set\_stepsize\_jitter(const double j) | stan::mcmc::base\_hmc< M, dense\_e\_point, dense\_e\_metric, expl\_leapfrog, BaseRNG > | inlinevirtual |
| set\_T(const double t) | stan::mcmc::base\_static\_hmc< M, dense\_e\_point, dense\_e\_metric, expl\_leapfrog, BaseRNG > | inline |
| set\_window\_params(unsigned int num\_warmup, unsigned int init\_buffer, unsigned int term\_buffer, unsigned int base\_window, std::ostream \*e=0) | stan::mcmc::stepsize\_covar\_adapter | inline |
| stepsize\_adaptation\_ | stan::mcmc::stepsize\_covar\_adapter | protected |
| stepsize\_covar\_adapter(int n) | stan::mcmc::stepsize\_covar\_adapter | inline |
| T\_ | stan::mcmc::base\_static\_hmc< M, dense\_e\_point, dense\_e\_metric, expl\_leapfrog, BaseRNG > | protected |
| transition(sample &init\_sample) | stan::mcmc::adapt\_dense\_e\_static\_hmc< M, BaseRNG > | inlinevirtual |
| update\_L\_() | stan::mcmc::base\_static\_hmc< M, dense\_e\_point, dense\_e\_metric, expl\_leapfrog, BaseRNG > | inlineprotected |
| write\_sampler\_param\_names(std::ostream &o) | stan::mcmc::base\_static\_hmc< M, dense\_e\_point, dense\_e\_metric, expl\_leapfrog, BaseRNG > | inlinevirtual |
| write\_sampler\_params(std::ostream &o) | stan::mcmc::base\_static\_hmc< M, dense\_e\_point, dense\_e\_metric, expl\_leapfrog, BaseRNG > | inlinevirtual |
| write\_sampler\_state(std::ostream \*o) | stan::mcmc::base\_hmc< M, dense\_e\_point, dense\_e\_metric, expl\_leapfrog, BaseRNG > | inlinevirtual |
| z() | stan::mcmc::base\_hmc< M, dense\_e\_point, dense\_e\_metric, expl\_leapfrog, BaseRNG > | inline |
| z\_ | stan::mcmc::base\_hmc< M, dense\_e\_point, dense\_e\_metric, expl\_leapfrog, BaseRNG > | protected |
| ~adapt\_dense\_e\_static\_hmc() | stan::mcmc::adapt\_dense\_e\_static\_hmc< M, BaseRNG > | inline |
| ~base\_mcmc() | stan::mcmc::base\_mcmc | inlinevirtual |
| ~base\_static\_hmc() | stan::mcmc::base\_static\_hmc< M, dense\_e\_point, dense\_e\_metric, expl\_leapfrog, BaseRNG > | inline |


---

[ Stan Home Page ]

*© 2011–2014,
Stan Development Team.*
